# Ambient nitrogen dioxide pollution and spreadability of COVID-19 in Chinese cities

**DOI:** 10.1016/j.ecoenv.2020.111421

**Published:** 2021-01-15

**Authors:** Ye Yao, Jinhua Pan, Zhixi Liu, Xia Meng, Weidong Wang, Haidong Kan, Weibing Wang

**Affiliations:** a Department of Biostatics, School of Public Health, Fudan University, Shanghai 200032, China; b Department of Epidemiology, School of Public Health, Fudan University, Shanghai 200032, China; c Key Laboratory of Public Health Safety of Ministry of Education, Fudan University, Shanghai, China; d Department of Environmental Health, School of Public Health, Fudan University, Shanghai 200032, China; e Shanghai Key Laboratory of Meteorology and Health, Shanghai 200032, China

**Keywords:** SARS-CoV-2, COVID-19, Ambient nitrogen dioxide, Impact factors

## Abstract

This study aims to explore the relationship between ambient NO_2_ levels and the transmission ability (basic reproductive number, R_0_) of COVID-19 in 63 Chinese cities. After adjustment for temperature and relative humidity, R_0_ was positively associated with NO_2_ concentration at city level. The temporal analysis within Hubei province indicated that all the 11 Hubei cities (except Xianning City) had significant positive correlations between NO_2_ concentration (with 12-day time lag) and R_0_ (r > 0.51, *p* < 0.005). Since the association between ambient NO_2_ and R_0_ indicated NO_2_ may increase underlying risk of infection in the transmission process of COVID-19. In addition, NO_2_ is also an indicator of traffic-related air pollution, the association between NO_2_ and COVID-19′s spreadability suggest that reduced population movement may have reduced the spread of the SARS-CoV-2.

## Highlights

● The basic reproductive number of COVID-19 was positively associated with NO_2_ after adjustment for temperature and relative humidity.
● Reduced population movement (NO_2_ decreases) may reduce the spread of the COVID-19.

## 1 Introduction

The COVID-19 pandemic has highlighted the importance of international solidarity and unity in the face of a dire global health and economic crisis. The pandemic, which was first reported in December 2019 in Wuhan, China, has caused 6,757,764 confirmed cases worldwide as of Jul 31, 2020, with 88,077 cases reported in China (NHC, 2020). Although massive intervention measures (*e.g.*, shutting down cities, extending holidays, and travel bans) have been implemented in China and many other countries, the spread of the disease is unlikely to be stopped worldwide in the near future. No effective vaccines or antiviral drugs have been clinically approved so far. Our current understanding of the factors that impact SARS-CoV-2 transmission is still limited.

Environmental factors are associated with the seasonality of respiratory-borne disease epidemics (Sooryanarain and Elankumaran, 2015). Some research has investigated both indoor and outdoor environmental nitrogen dioxide (NO_2_) pollution exposure to individuals (Salonen et al., 2019). Previous cross-sectional and cohort research has provided evidence that ambient NO_2_ exposure had longitudinal effects on growth in lung function (Molter et al., 2013), causing pulmonary insufficiency (*e.g*., lung volume, expiratory flow). In addition, previous studies have suggested that ambient NO_2_ exposure may play a role in the phenotypes of respiratory diseases including but not limited to influenza (Huang et al., 2016), asthma (Weinmayr et al., 2010), and severe acute respiratory syndrome (Kan et al., 2005). For example, NO_2_ might increase adults’ susceptibility to viral infections (Goings et al., 1989). Exposure to high levels of NO_2_ before the start of a respiratory viral infection is associated with the severity of asthma exacerbation (Chauhan et al., 2003).

Recently, a European study found that 78% of COVID-19 fatalities were located in five regions that showed the highest concentrations of NO_2_ (Ogen, 2020). This finding indicates that long-term NO_2_ exposure may be an important risk factor for COVID-19 fatality. However, Contini et al. (Contini and Costabile, 2020) discussed the relationships between atmospheric parameters and COVID-19 prevalence or fatality are influenced by several confounding factors, which made difficult to interpret correlations that are not indicating necessarily a cause-effect relationship in the description study. Although it’s an inevitable limitation in our description study, our study aims to thoroughly explore the influence of NO_2_ on COVID-19 transmission and to try to acquire more solid results with potential confounders adjusted.

## 2 Theory/calculation

In this study, we aim to assess the associations between ambient NO_2_ levels and the spreadability of COVID-19 across 63 Chinese cities, and we provide information to facilitate the further prevention and control of COVID-19.

## 3 Methods

### 3.1 Data collection

We collected COVID-19 confirmed case information reported by the National Health Commission of the People’s Republic of China (WHO, 2020) and Health Commission of Hubei Province (http://wjw.hubei.gov.cn/bmdt/ztzl/fkxxgzbdgrfyyq/). Guidelines on the diagnosis and treatment of patients were defined according to the fourth version of the guidelines (issued on January 27, 2020). The clinical criteria for diagnosis were to meet any two of the three remaining clinical criteria (*i.e*., fever, radiographic findings of pneumonia, and normal or reduced white blood cell count or reduced lymphocyte count in the early stage of illness). An epidemiological criterion was added (*e.g.*, linkage with a confirmed COVID-19 case) (NHC, Zhang et al., 2020). The population movement in cities outside Hubei from the same period was obtained from Baidu Qianxi data (https://qianxi.baidu.com/2020/), and we used migration index and travel intensity to describe the movement. We obtained hourly concentrations of various air pollutants, including sulfur dioxide (SO_2_), NO_2_, carbon monoxide (CO), ozone (O_3_), fine particulate matter (PM_2.5_), and inhalable particulate matter (PM_10_). These data came from 63 cities (cities in China with more than 50 confirmed COVID-19 cases as of February 10, 2020) and ranged from January 1, 2020 to February 8, 2020. The data were acquired from the National Urban Air Quality Publishing Platform (http://106.37.208.233:20035/), which is administered by China’s Ministry of Environmental Protection. Daily concentrations of these air pollutants were calculated as the average of at least 18 (75%) hourly concentrations for all state-controlled stations, and then the values for each city were calculated as the average among all valid stations within the city limits. We calculated the average daily concentrations of these air pollutants in all 63 Chinese cities. In addition, the average of the annual NO_2_ concentrations from 2016 to 2019 was obtained to estimate the long-term exposure level. Other meteorological data including daily mean temperature and relative humidity were collected from the China Meteorological Data Sharing Service System.

### 3.2 Basic reproductive number

The reproductive number (R_0_), the average number of individuals infected by an initial infectious individual in a completely susceptible population, is fundamental to understanding disease transmission. We calculated R_0_ for 63 Chinese cities with more than 50 cases as of February 10, 2020 (the COVID-19 peak period in China), including 12 and 51 cities inside and outside Hubei, respectively. We used the method introduced by Aaron et al. to estimate R_0_ (King et al., 2017). First, we constructed a linear regression model to estimate the relevant coefficient. Second, we obtained R_0_ by combining the coefficients obtained from the previous step with the average incubation and confirmation periods. We assigned the average values of the incubation period and the mean course from case infection to confirmation as 7 and 3.8 days, respectively. These values were obtained in previous mathematical research (Pan et al., 2020). All calculations were completed in R software version 3.6.1 (R Foundation for Statistical Computing).

### 3.3 Testing for mediation

Mediation is a hypothesized causal chain in which one variable affects a second variable that, in turn, affects a third variable (Lederer et al., 2019). The relationship between NO_2_ concentration and R_0_ of COVID-19 may be mediated by population density or other air pollutants, such as city population and city area. Those mediators may indirectly affect the R_0_ value of COVID-19 by modulating the NO_2_ concentration, thus affecting the spread of COVID-19. In this study, we used mediation analysis to explore whether these factors were mediators of the relationship between NO_2_ and R_0_ of COVID-19, and we used bootstrapping to estimate standard error while testing the significance of these mediating effects.

### 3.4 Statistical analysis

We conducted a cross-sectional analysis to examine the associations of NO_2_ with R_0_ of COVID-19. We also conducted a longitudinal analysis to examine the temporal associations (with daily data points) of NO_2_ with R_0_ in cities inside Hubei Province since the date when they had enough confirmed cases to acquire stable daily R_0_ values. The other covariates, including health policies, were quite similar throughout Hubei Province. When examining the correlation between NO_2_ and R_0_ of COVID-19, we estimated the associations of NO_2_ concentration with R_0_ both inside and outside Hubei province (r & *p*) in the same period by using multiple linear regression models after controlling for temperature and relative humidity (as covariates in the regression model) separately. Then, we used meta-analysis to pool the estimates of the specific associations of NO_2_ concentration with R_0_ (meta χ^2^ & *p*). We also examined the corresponding temporal associations between NO_2_ and R_0_ of COVID-19 across the different cities inside and outside Hubei Province using multiple linear regression models after controlling for temperature and relative humidity separately. The change of R_0_ per 10 μg/m^3^ increase in NO_2_ pollution was calculated. Given that associations between NO_2_ and COVID-19 prevalence are influenced by several confounding factors, we further examined the associations of NO_2_ with the R_0_ of COVID-19 with adjustment for density of population, GDP per capita and hospital beds per capita in the main model. Additionally, residual analysis was conducted to test the reliability of correlation. And principal component analysis was used to explore the relevance of various factors. The χ^2^ values and corresponding *p* values were obtained by combining the correlations together using the meta-analysis method. This part of the statistical analysis was conducted with MATLAB R2019a. A *p* value of less than 0.05 was considered to indicate statistical significance.

## 4 Results

### 4.1 Temporal and spatial distributions of NO_2_

**Fig. 1:**
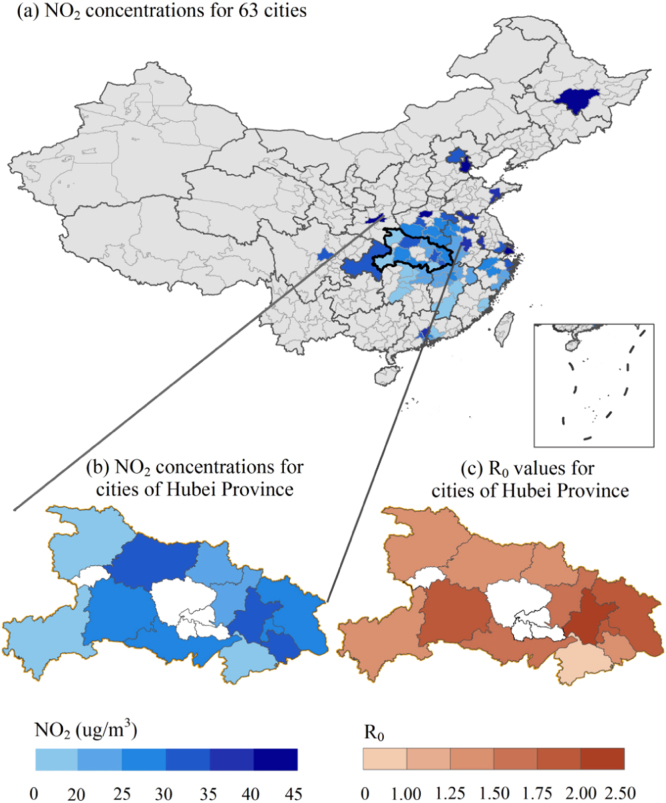
Spatial distribution of average nitrogen dioxide concentration and spreadability of COVID-19. This map of China shows the spatial distribution of the average nitrogen dioxide concentration in 63 Chinese cities from January 1, 2020 to February 8, 2020. The zoomed-in view of Hubei Province compares the spatial trend of the average nitrogen dioxide concentration (gradient blue map, bottom left) with that of the basic reproductive number R_0_ (gradient brown map, bottom right). (For interpretation of the references to color in this figure legend, the reader is referred to the web version of this article.)

**Fig. 2:**
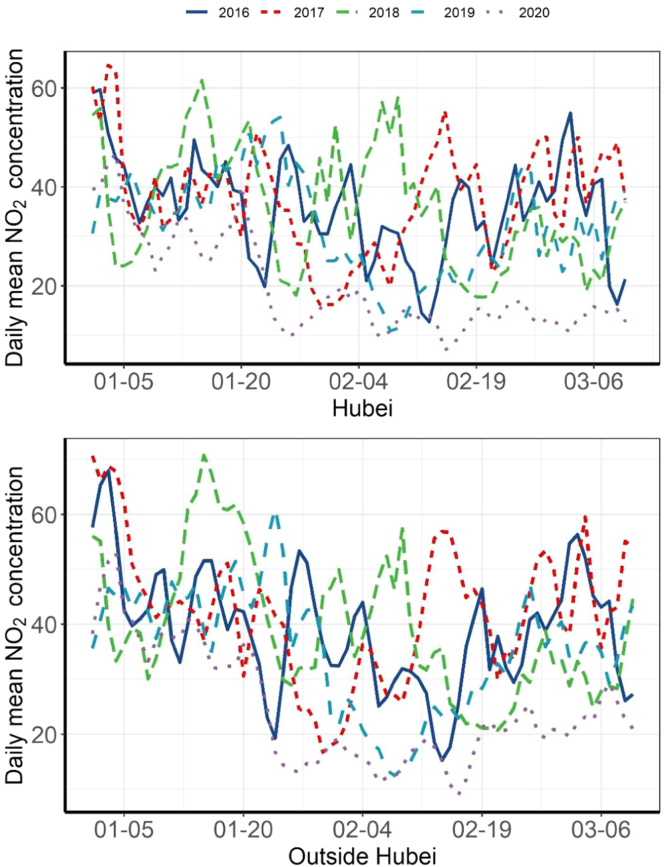
Daily variation of mean NO_2_ concentration in 2016–2020. The above panel shows the change of the average NO_2_ concentration of 12 cities in Hubei Province from January 1 to March 10, 2016–2020. The following panel shows the change of the average NO_2_ concentration of 51 cities in Hubei Province from January 1 to March 10, 2016–2020, in which the lines of different colors represent different annual shares. (For interpretation of the references to color in this figure legend, the reader is referred to the web version of this article.)

Among the 63 investigated cities, the mean±standard deviation and range of NO_2_ concentration and R_0_ were (27.9 ± 8.3 ug/m^3^, 10.7–53.0 ug/m^3^) and (1.4 ± 0.3, 0.6–2.5), respectively. The cities with the three highest R_0_ values were Wuhan, Huanggang, and Yichang, which are all in Hubei Province. The similarity of the spatial distributions between R_0_ and NO_2_ suggests a relationship between R_0_ and NO_2_ concentration (Fig. 1). No matter Hubei Province or outside of Hubei Province, the daily concentration trend of NO_2_ from January to march in 2016–2019 is almost the same, but it is obvious that the daily concentration of NO_2_ in 2020 is lower than that in other years, especially after January 23, 2020 (Fig. 2), which may be due to the closure of Wuhan city in Hubei.

### 4.2 Correlation analysis of spatial distribution

**Fig. 3:**
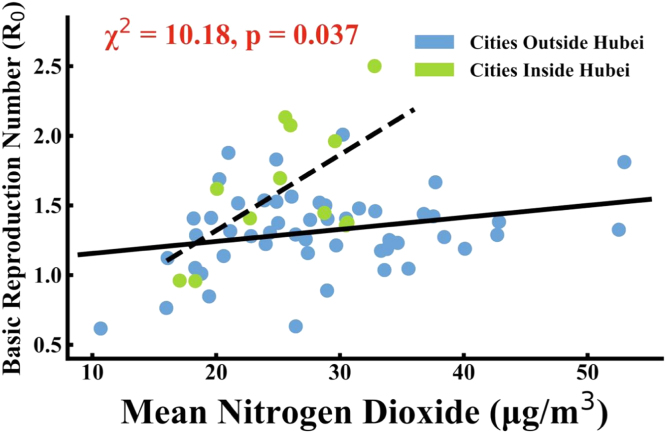
Nitrogen dioxide concentration and spreadability of COVID-19, divided by area. The basic reproductive number R_0_ was positively associated with NO_2_ (meta χ^2^ = 10.18, *p* = 0.037) in cities outside Hubei (blue points, 51 cities, r = 0.29, *p* = 0.046, solid line) and cities inside Hubei (green points, 12 cities, r = 0.51, *p* = 0.13, dashed line). We controlled the effects from temperature and relative humidity in the multiple linear regression models. (For interpretation of the references to color in this figure legend, the reader is referred to the web version of this article.)

The scatter diagram of R_0_ and NO_2_ distributions (Fig. 3) shows that R_0_ tends to increase with NO_2_ concentration, suggesting a positive correlation between R_0_ and NO_2_ concentration. The cross-sectional analysis indicates that, after adjustment for temperature and relative humidity, R_0_ was positively associated with NO_2_ concentration at city level (meta χ^2^ = 10.18, *p* = 0.037) (Fig. 3). Additionally, we further examined the associations of NO_2_ with the R_0_ of COVID-19 adjusted for density of population, GDP per capita, hospital beds per capita separately in the main model, and we found that none of the three covariates would affect the significant positive association between NO_2_ with R_0_. In the following stratified analysis, a significant association was confirmed in cities outside Hubei (r = 0.29, *p* = 0.046), whereas the trend observed in cities inside Hubei was not significant (r = 0.51, *p* = 0.130) (Fig. 3). For every 10 μg/m^3^ increase in NO_2_, R_0_ increased by 0.12 (0.01–0.23) and 0.52 (−0.20–1.25), respectively. We did not find significant associations of temperature or relative humidity with R_0_ of COVID-19 (meta χ^2^ = 4.62, *p* = 0.370 and meta χ^2^ = 1.63, *p* = 0.800, respectively).

**Fig. 4:**
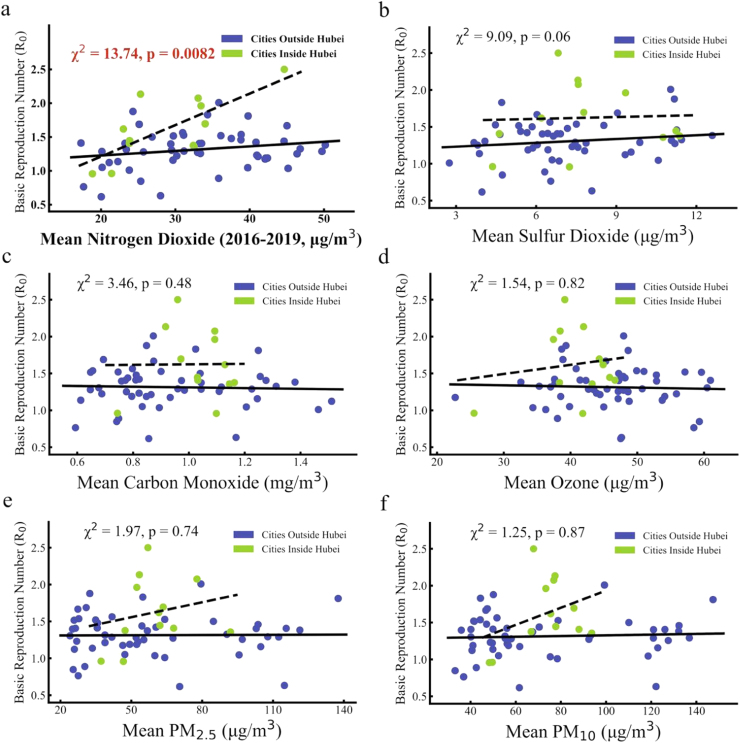
Associations of mean concentrations of nitrogen dioxide (2016–2019) and other air pollutants with spreadability of COVID-19, divided by area. (a) The basic reproductive number R_0_ was positively associated (meta χ^2^ = 13.74, *p* = 0.0082) with the average NO_2_ value from 2016 to 2019. (b)–(f) There were no significant associations between other air pollutants (SO_2_, CO, O_3_, PM_2.5_, and PM_10_) and R_0_ (meta χ^2^ < 9.09, *p* > 0.06). We controlled the effects from temperature and relative humidity in the multiple linear regression models.

In addition, we found that R_0_ was positively associated with the average NO_2_ value from 2016 to 2019 (meta χ^2^ = 13.74, *p* = 0.008; Fig. 4a) with adjustment for temperature and relative humidity. Because the average NO_2_ value from 2016 to 2019 was significantly associated with that in early 2020 (r = 0.85, *p* < 0.0001), it is difficult to determine which factor is dominant in COVID-19 transmission. Moreover, the other investigated air pollutants (SO_2_, CO, O_3_, PM_2.5_, and PM_10_) had no significant associations with R_0_ (meta χ^2^ < 9.09, *p* > 0.06; Fig. 4b–f). Furthermore, in order to avoid potential population movement effects in our study, which could decrease both NO_2_ and R_0_, we collected reduced population movement data from 51 cities outside Hubei in the same period. We re-calculated NO_2_–R_0_ associations including the population movement as a covariate, and we found that the NO_2_ was still significantly correlated with R_0_ of COVID-19 outside Hubei (r = 0.32, *p* = 0.024).

### 4.3 Temporal correlation analysis

**Fig. 5:**
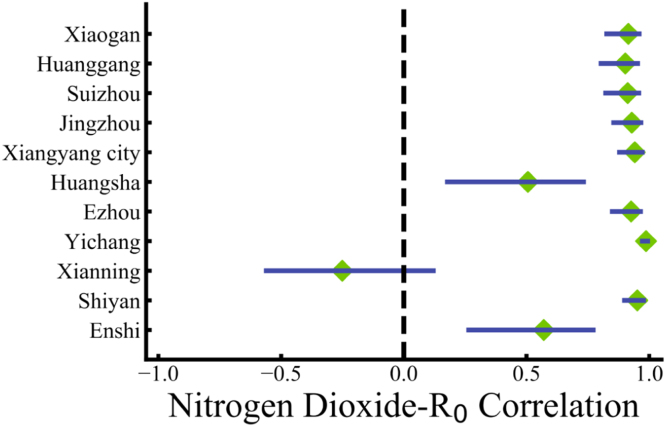
Temporal correlation between nitrogen dioxide concentration and spreadability of COVID-19 in Hubei. Temporal correlation between NO_2_ concentration and R_0_ in 11 cities in Hubei. Except for Xianning, all of those cities had significant positive correlations (r > 0.51, *p* < 0.005) between NO_2_ (with 12-day time lag) and daily R_0_ (normalized based on Wuhan’s daily R_0_).

We calculated the daily R_0_ values of 11 cities in Hubei (except Wuhan) from January 27 to February 26, 2020 (there were few COVID-19 confirmed cases in these cities afterwards) and normalized them based on Wuhan’s daily R_0_ value to eliminate the effects of other covariates. We found that 11 Hubei cities (except Xianning City) had significantly positive correlations between NO_2_ concentration (with 12-day time lag) and R_0_ (r > 0.51, *p* < 0.005), suggesting a positive association between daily NO_2_ concentration and COVID-19 spreadability on the temporal scale (Fig. 5). The same conclusion was reached for other time lag settings, but the most significant value was obtained with a delay of 12 days. The results of residual analysis and principal component analysis were shown in Fig. S1 and Fig. S2, respectively.

### 4.4 Testing for mediation

To eliminate the effects of city population and city area on the relationship between NO_2_ concentration and R_0_ value, we applied a mediation analysis to verify whether more densely populated cities had both greater R_0_ and NO_2_ concentration values. After adjustment for temperature and relative humidity, the mediation analysis found insignificant direct and indirect effects of city population and city area on R_0_ (z = −1.43, *p* = 0.15 & z = −0.24, *p* = 0.800 and z = −0.46, *p* = 0.650 & z = 1.15, *p* = 0.250, respectively). Thus, there were no apparent mediation effects between city population, city area, NO_2_, and R_0_. City population and city area did not influence the association between NO_2_ concentration and R_0_.

## 5 Discussion

This study explored the association between environmental factors and COVID-19 transmission. To our knowledge, little research has been done on the relationship between ambient air pollution and COVID-19 transmission. Our results show a significant association between NO_2_ exposure and R_0_, suggesting that ambient NO_2_ may contribute to the spreadability of COVID-19. To prevent city population and city area from affecting the relationship between NO_2_ concentration and R_0_ level, we applied a mediation analysis to verify whether more densely populated cities have both greater R_0_ values and higher NO_2_ concentrations. The results showed that city population and city area did not influence the association between NO_2_ concentration and R_0_ level.

Although the closures of cities throughout Hubei occurred at approximately the same time point: the other cities of Hubei were locked down no longer than 1–2 days later than Wuhan City, the effect of the lockdown measure in different cities (*e.g.* cities with busy traffic *vs.* small rural cities) was not expected to have the same influence on the association between NO_2_ and COVID-19 transmission. Multiple impact factors (the population density of the city, the typical road traffic and commercial exchanges, *etc*.) may still have confounded the association in the current analysis, but we have controlled for as many factors as possible to reduce confounding and solid our results, including the density of population, GDP per capita and hospital beds per capita. Previous studies also have suggested that the increased spreadability resulting from NO_2_ exposure might be caused by the effects of NO_2_ on host defenses that prevent viral spread (Becker and Soukup, 1999). Chen et al. (2007) found that exposure to NO_2_ may harm to humans’ health by interacting with the immune system; besides, Mills et al. (2015) observed that short-term exposure to NO_2_ had increased the hospital admission rates for a range of respiratory diseases in different age groups. Therefore, we speculated NO_2_ has potential ability to contribute in the infection process of COVID-19 directly.

In addition, NO_2_ emissions primarily come from burning fossil fuels (diesel, gasoline, coal), resulting in automobile and smokestack exhaust, the latter of which can be produced by electricity generation. Therefore, changes in NO_2_ levels can be used to indicate changes in human activity and population movement due to the lockdown of cities. For example, we can see that since January 23, 2020, the daily average concentration of NO_2_ after the closure of Wuhan is obviously lower than that of the same period in previous years (Fig. 2). Besides, it is well known that the spread of respiratory virus is through contact (direct or indirect *via* fomites) or through contaminated droplets emitted by cough, sneeze, respiration and speaking of infected individuals, both of which are related with human contact, social distance and population movement. Plus, NO_2_ is as an indicator of traffic-related air pollution, the association between NO_2_ and R_0_ of COVID-19 may be explained by the relationship between viral spread and population movement. Of course, further investigations are warranted to provide additional details and illustrate this mechanism.

Our study has some limitations: first, the averaging of NO_2_ concentrations across cities likely resulted in an unknown degree of exposure misclassification, given the spatial variability and traffic-dependence of NO_2_ and the potential for indoor exposure. Second, R_0_ could be highly variable and is influenced by a variety of factors, including not only the previously mentioned mitigation efforts but also the comprehensiveness of case identification. Third, for the lack of corresponding data of NO, we did not explore the association between primary pollutant NO and the transmission ability of COVID-19. Given the ecological nature of this study, other city-level factors, such as the implementation ability of COVID-19 control policy, urbanization rate, and availability of medical resources, may affect the transmissibility of COVID-19 and confound our findings. Future studies should develop individual-based models with high spatial and temporal resolution to assess the correlations between air pollution and the epidemiologic characteristics of COVID-19. The mechanisms between NO_2_ and the transmission of COVID-19 disease still require further research, besides, the spread of COVID-19 could be affected by many factors. We also believe that there is likely to have interaction of environmental factors and NPIs, which deserves further analysis.

## Role of the funding source

The funders of the study had no role in study design, data collection, data analysis, data interpretation, or writing the report. The corresponding author had full access to all of the study’s data and takes final responsibility for the decision to submit for publication.

## Funding

This work was supported by the Bill & Melinda Gates Foundation, Seattle, WA [Grant No. INV-006277] and the Fudan University Research Project on COVID-19 Emergency [Grant No. IDF201007].

## CRediT authorship contribution statement

**Ye Yao:** Data curation, Conceptualization, Methodology, Software. **Jinhua Pan:** Data curation, Writing - original draft. **Zhixi Liu:** Data curation, Writing - original draft. **Xia Meng:** Writing - original draft. **Weidong Wang:** Data curation, Resources. **Haidong Kan:** Supervision and Writing - reviewing & editing. **Weibing Wang:** Supervision and Writing - reviewing & editing. All authors critically reviewed and approved the final version of the manuscript. The corresponding authors are responsible for ensuring that the descriptions are accurate and agreed by all authors.

## Declaration of Competing Interest

The authors declare that they have no known competing financial interests or personal relationships that could have appeared to influence the work reported in this paper.
