# Supplementary material for: Ambient nitrogen dioxide pollution and spreadability of COVID-19 in Chinese cities
Source: Ecotoxicol Environ Saf. 2021 Jan 15;208:111421. doi: 10.1016/j.ecoenv.2020.111421 (PMC7524685; doi:10.1016/j.ecoenv.2020.111421)
Supplement: Supplementary file 1 — Supplementary material. [file mmc1.doc]

Ambient nitrogen dioxide pollution and spread ability of COVID-19 in Chinese cities

Ye Yao^1†^ Ph.D, Jinhua Pan^2†^ M.Sc., Zhixi Liu^2†^ B.Med., Xia Meng^4†^ Ph.D., Weidong Wang^4^ B.Med., Haidong Kan^3,4*^ Ph.D., Weibing Wang^2,3*^ Ph.D.,

^1^Department of Biostatics, School of Public Health, Fudan University, Shanghai 200032, China

^2^Department of Epidemiology, School of Public Health, Fudan University, Shanghai 200032, China

^3^Key Laboratory of Public Health Safety of Ministry of Education, Fudan University, Shanghai, China

^4^Department of Environmental Health, School of Public Health, Fudan University, Shanghai 200032, China

^†^Dr. Yao, Ms. Pan, Ms. Liu and Dr. Meng contributed equally to this letter.

* Corresponding authors:

Dr. Weibing Wang, School of Public Health, Fudan University, Shanghai 200032, China, Email: wwb@fudan.edu.cn

Dr. Haidong Kan, School of Public Health, Fudan University, Shanghai 200032, China, Email: kanh@fudan.edu.cn

Content:

1. Figure S1. Residual analysis diagram

2. Figure S2. Principal component analysis diagram


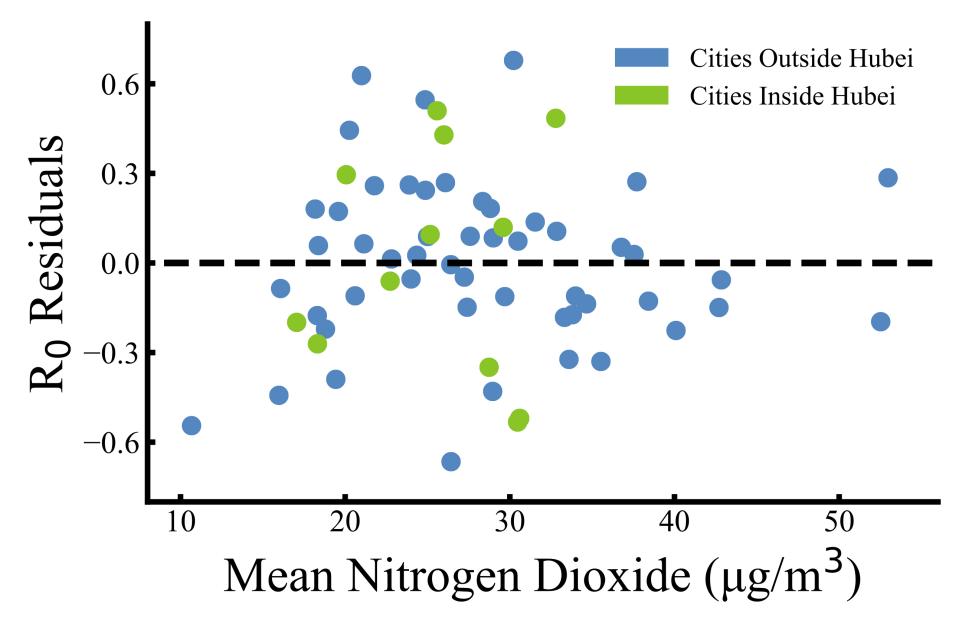


**Figure S1. Residual analysis diagram**


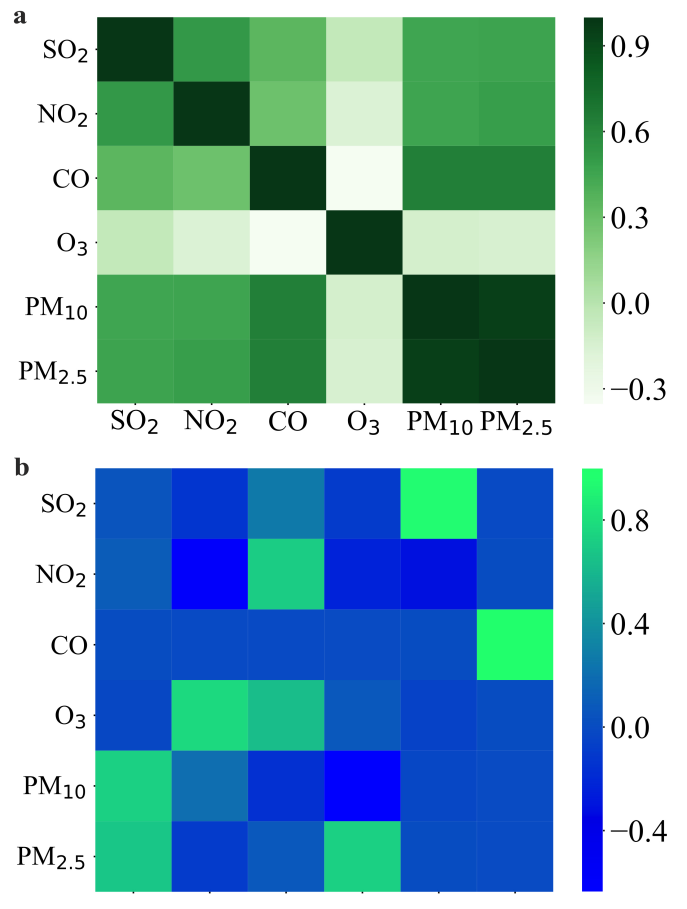


**Figure S2. Principal component analysis diagram**

**Panel a:** the correlation diagram exhibits the relationship between environmental factors; **Panel b:** the rows of coeff contain the coefficients for the six ingredient variables, and its columns correspond to six principal components.
